# Prostate Cancer Metastatic to the Cervical Lymph Nodes

**DOI:** 10.1155/2015/263978

**Published:** 2015-03-04

**Authors:** Luis Sepúlveda, Tiago Gorgal, Vanessa Pires, Filipe Rodrigues

**Affiliations:** ^1^Urology Department, Trás-os-montes and Alto Douro Hospital Center, 5000-508 Vila Real, Portugal; ^2^Internal Medicine Department, Trás-os-montes and Alto Douro Hospital Center, 5000-508 Vila Real, Portugal

## Abstract

Prostate cancer is the most common cancer in men, often presenting with regional lymph node or bone metastasis and rarely with supradiaphragmatic lymph node involvement. Most metastatic cancers involving the cervical lymph nodes are from cancers of the upper aerodigestive tract. In this report, we describe two cases with cervical lymph node enlargement due to metastatic prostate cancer as the initial clinical presentation: a 43-year-old male, initially misdiagnosed with a tumor of the upper aerodigestive tract and an 87-year-old male with right lobe pneumonia and cervical lymph node enlargement, initially attributed to be an acute inflammatory lymph node reaction. To the best of our knowledge, there are less than 50 cases reported in the literature of adenocarcinoma of prostate metastatic to the cervical lymph nodes and only one case presenting in men younger than 45 years. The authors intend to highlight the importance of digital rectal exam and PSA test in case of persistent left cervical lymph node enlargement, including men younger than 45 years of age.

## 1. Introduction

In Europe, carcinoma of the prostate is the most common solid neoplasm and the second most common cause of cancer death in men [1, 2]. With a peak incidence between the ages of 70 and 74 years, less than 0.1% of all patients with prostate cancer are younger than 50 years of age [3]. The most common metastatic site is the lymph nodes, usually those of the pelvis and retroperitoneum.

Cervical lymph node enlargement in children and young adults is usually related to an inflammatory condition or lymphoma. In the elderly it can also reflect a manifestation of lymphoma or a metastatic dissemination from either squamous cell carcinoma or adenocarcinoma. Most cancers that present cervical lymphadenopathy are derived from head and neck malignancies involving the mucosal surfaces of the aerodigestive tract [4]. The most frequent nonhead or neck primary cancers metastatic to the cervical chain are lung, breast, and kidney [5]. Despite the high incidence and prevalence of prostate cancer, there are less than 50 case reports of metastatic involvement of the cervical lymph nodes and only one case presenting in men younger than 45 years [6–8].

This report describes 2 cases of prostate cancer metastatic to the cervical lymph nodes: a 43-year-old man and an elderly 87-year-old male, initially misdiagnosed, respectively, as primary neck cancer and inflammatory lymphadenopathies due to pneumonia.

## 2. Case Report 1

**Figure 1:**
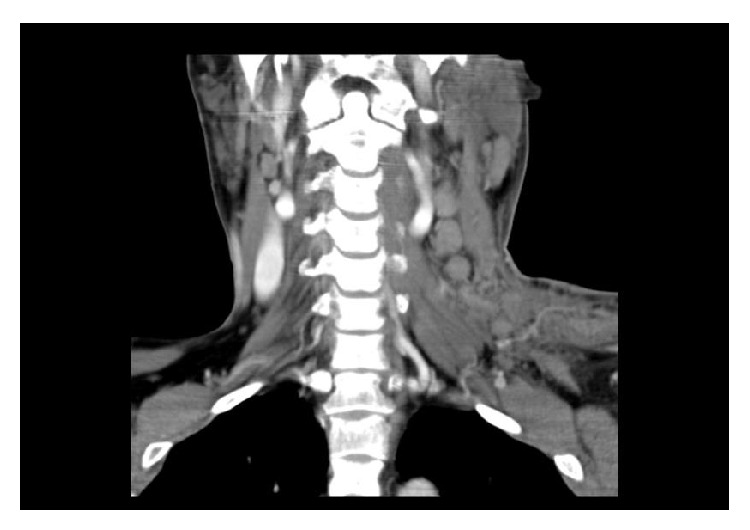
CT coronal reconstruction revealing the presence of diffuse left cervical lymphadenopathy.

**Figure 2:**
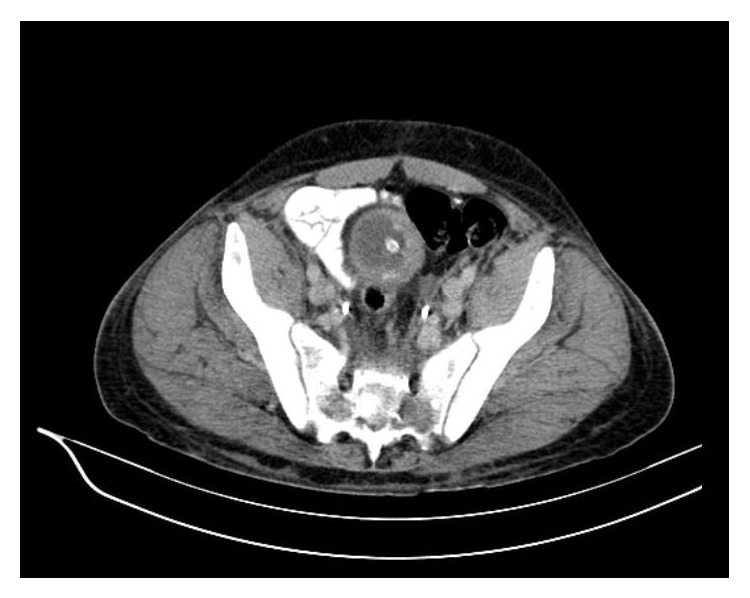
Pelvic CT demonstrating enlarged lymph nodes in the pelvis, more pronounced in the left iliac region.

**Figure 3:**
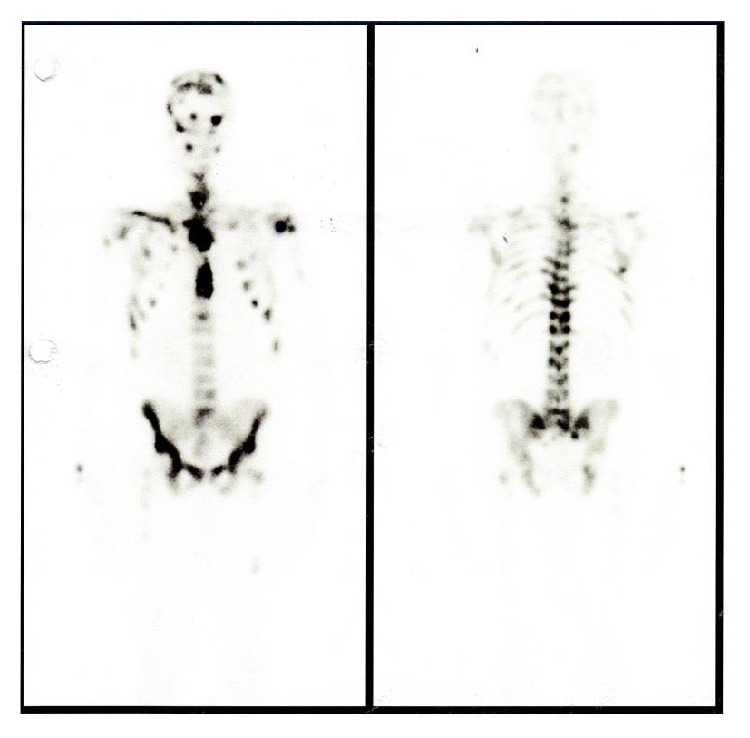
Tc-99m body scan revealing multiple skeletal lesions, confirming the previous tomography suspicion of axial, sternum, and cranium bone metastasis.

**Figure 4:**
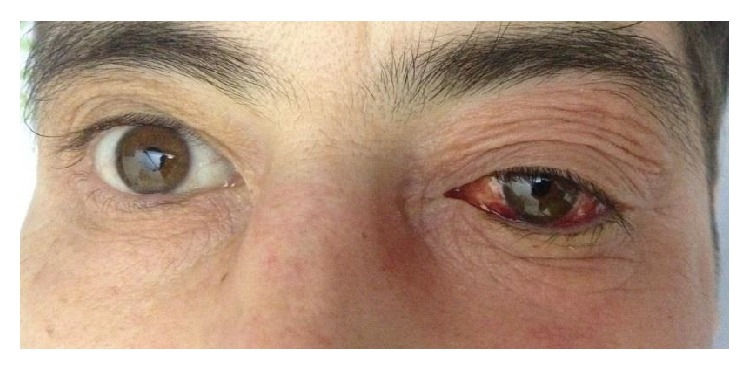
Left eye hyperemia and exophthalmos due to sphenoidal bone metastasis compressing the eye globe.

A 43-year-old Caucasian male was referred to Internal Medicine with complaints of left cervical mass, anorexia, and 10 kg of unintentional weight loss in 2 months. He denied any other subjective complaints, including difficulty in swallowing, breathing, or urinary symptoms. On examination, a nontender, firm mass with approximately 3 cm in diameter was present in the left cervical region, fixed to the underlying tissues. There were also nodes palpable in the left axillar and bilateral inguinal region. Subsequent computed tomography (CT) scan of the neck, thorax, and abdomen showed multiple adenopathies involving retroperitoneum, inguinal, iliac, left axillar and left cervical regions, signs of pronounced bone metastasis, and bilateral hydronephrosis (Figures 1 and 2). A biopsy of the neck lymphadenopathy was performed and interpreted as probable adenocarcinoma, strongly positive for prostate specific antigen in immunohistochemical study. Full blood count, biochemical investigation, and prostate specific antigen (PSA) were abnormal: creatinine 3.9 mg/dL (reference range (RR) 0.7–1.4 mg/dL), blood urea nitrogen (BUN) 93 mg/dL (RR: <50 mg/dL), PSA 583 ng/mL, alkaline phosphate 1592 U/L (RR: 40–130 U/L), and LDH 1086 U/L (RR: 135–225 U/L). Following consultation by urology, a digital rectal exam revealed a firm, fixed, and enlarged prostate, highly suspicious of prostate cancer. The patient denied family history of malignant neoplasms, including prostate cancer, and was never subjected to prostate exam or PSA test. He was then submitted to urgent bilateral ureteral stenting due to obstructive acute renal failure secondary to invasion of the ureteral meatus by prostate carcinoma. Transrectal ultrasonography guided prostate biopsy revealed bilateral adenocarcinoma with a Gleason score 3 + 5 = 8. The Tc-99m whole body scan confirmed the previous tomography suspicion of axial, sternum, and cranium bone metastasis (Figure 3). The patient initiated complete androgen blockage and zoledronic acid. Three months later his cervical lymph node enlargement had regressed and serum PSA had decreased to 3.48 ng/mL. Unfortunately 5 months after initiation of hormonal treatment the patient presented with complaints of left red eye, exophthalmos, and sudden decrease in visual acuity (Figure 4). Head CT showed an intracranial mass originating in the sphenoid bone and compressing the left eye ball. PSA had increased to 80.82 ng/mL and testosterone remained within castration level (<50 ng/dL). Chemotherapy with docetaxel associated with prednisolone was initiated after failure of standard hormonal manipulations and the patient presented clinical improvement with reduction of exophthalmos and pain complaints. After 5 cycles of docetaxel the PSA level increased to 370 ng/mL. The docetaxel, prednisolone, and zoledronic acid therapy was then substituted for denosumab and cabazitaxel, with good clinical and analytical response (current PSA level of 37 ng/mL).

## 3. Case Report 2

An 87-year-old male was admitted to the Hospital due to right lower lobe pneumonia. During the physical examination, several palpable bilateral supraclavicular adenopathies were noticed as well as mild alterations in respiratory sounds in the base of the right lung. Chest X-ray revealed an enlarged mediastinum and paratracheal tissues and signs of right lower lobe pneumonia. Full blood count and biochemical investigation presented mild leukocytosis (12.100 ×10^3^/*μ*L), hypochromic microcytic anemia, renal failure (creatinine 2 mg/dL, BUN 121 mg/dL), and normal values of alkaline phosphate and lactate dehydrogenase. His past medical history included atrial fibrillation, congestive heart disease, chronic obstructive pulmonary disease, and a stroke one year before. The patient had a past history of transurethral resection of prostate in a foreign country, from which there was no access to further data.

**Figure 5:**
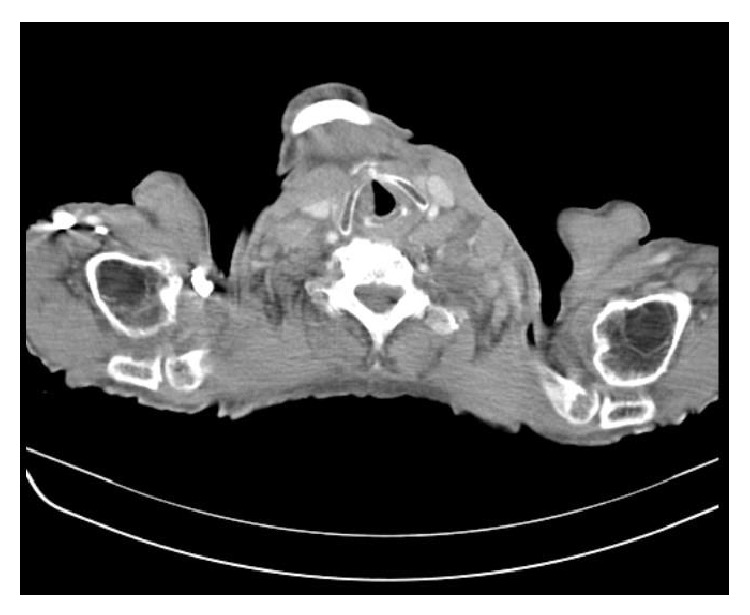
Cervical CT demonstrating multiple enlarged lymph nodes in the paratracheal, subcarinal, bilateral supraclavicular, and cervical regions.

The patient started on antibiotics, fluids reposition, and clinical surveillance, in accordance to the initial diagnosis of right lower lobe pneumonia and inflammatory cervical lymph node enlargement. A chest CT scan (Figure 5), performed one week after admittance, showed multiple enlarged lymph nodes in the paratracheal, retrocaval, subcarinal, bilateral supraclavicular, and cervical regions. There were no signs of pulmonary consolidation and clinically the patient improved greatly after the antibiotics. Due to persistence of lymphadenopathy, a percutaneous lymph node biopsy was performed as follows: histology and immunohistochemical study revealed probable adenocarcinoma, strongly positive for prostate specific antigen. The digital rectal exam revealed an enlarged, firm, and fixed prostate, highly suspicious of prostate cancer locally invasive. The PSA value was 1224 ng/mL, with normal calcium, lactate dehydrogenase, and alkaline phosphatase. Renal function was also normal. Ultrasound guided prostate biopsy was omitted and the patient initiated complete androgen blockage with leuprolide and bicalutamide.

**Figure 6:**
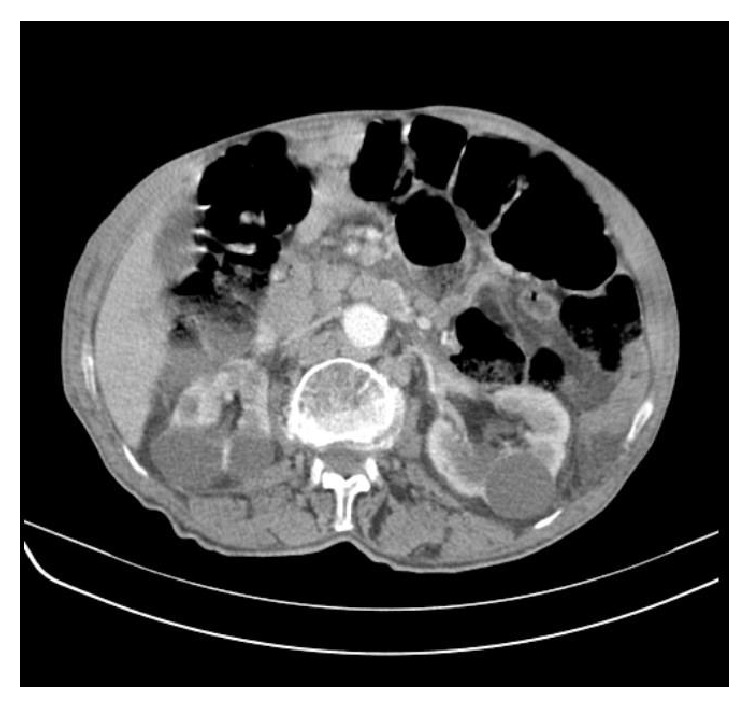
Abdominal CT demonstrating multiple enlarged lymph nodes in the retroperitoneum. The patient also presented bilateral renal cysts.

Further study with bone scintigraphy and pelvic CT revealed multiple pelvic and retroperitoneal lymphadenopathies (Figure 6), bone metastasis in vertebral body of T5 and left ischium, and absence of visceral metastasis. At that time the patient was asymptomatic.

Six months after initiation of complete androgen blockage the patient remained symptom-free with a PSA value of 2.2 ng/mL and presented partial regression of the mediastinum and supraclavicular lymph node enlargement.

## 4. Discussion

Usually, prostate cancer spreads primarily to the regional lymph nodes and bones, followed by lung, bladder, liver, and adrenal gland. Cervical lymph node involvement in prostate cancer is rare and is almost uniformly associated with widespread metastatic disease in patients over 45 years of age. The reported incidence varies between 0.28 and 0.4% in most series, with only one case in the literature presenting in a man younger than 45 years of age [9, 10].

Batson have postulated that head and neck metastases from prostate cancer occur due to hematogenous spread via the vertebral venous system (or Batson's plexus) [11]. However the hematogenous dissemination fails to explain the predilection of this carcinoma to metastasize to the left cervical region, whilst right side involvement is extremely uncommon. Prostate is richly supplied by lymphatics which drain into obturator-hypogastric and presacral nodes and from these to the iliac, paraaortic, cisterna chyli, and thoracic duct. Finally, the lymphatic drainage enters the systemic blood circulation via the left subclavian vein. Some authors postulated that tumor cells can lodge in the left cervical nodes by retrograde spread due to the proximity of these nodes with the point-of-entry of the thoracic duct into the left subclavian vein [12]. The authors believe that in the first case the cervical metastasis was due to lymphatic spread, as the pattern of lymph node invasion in the neck was limited to the left side. In the second case, the bilateral involvement of cervical lymph nodes is more favorable of a hematogenous spread.

Most previous case reports and small series of case analyses document relatively poor prognosis after such a presentation, with mean survival between 19.8 and 29.7 months [12, 13]. However, there is a case report of a patient with cervical lymphadenopathy being symptom-free for 9 years [14].

Early onset of prostate cancer, as presented in case report 1, differs in many ways from prostate cancer in elderly men (case report 2). Usually, these men present higher cause-specific mortality, have a strong genetic component, and suffer longer from treatment related side effects due to their extended life expectancy. The lack of significant number of patients with early onset of prostate cancer poses a unique challenge in terms of clinical research and genomic investigation. So far, little is known about early detection or management of prostate cancer in young patients (aged <55 years), due to the severe lack of randomized controlled trials regarding this matter. Most authors believe these cancers represent a distinct phenotype in prostate cancer, etiologically, and clinically, and therefore clear guidelines are needed for appropriate management and consistency of care [15, 16].

These case reports present different confounding factors responsible for error or delay in diagnosing a prostate cancer with cervical lymphadenopathies. In the first case, the absence of urinary complaints, the patient's age, and absence of ethnical or family risk factors made the hypothesis of prostate cancer highly unlikely. The main differential diagnosis included lymphoma and metastatic neoplasm of the upper aerodigestive tract. In the second case, the confounding factor was the presence of a simultaneous respiratory infection. The pneumonia has misled the medical staff into attributing an inflammatory reaction as the cause to lymph node enlargement. The authors would like to highlight the importance of digital rectal exam, almost pathognomonic in both cases.

These case reports illustrate the need of digital rectal exam and measurement of serum prostate specific antigen in adult males with persistent left cervical lymphadenopathy, even in patients younger than 45 years old. These exams are important to establish a timely diagnosis of prostate cancer, to institute the appropriate treatment and ensure the best possible prognosis.
